# Technological infrastructure, sleep, and rest-activity patterns in a Kaqchikel Maya community

**DOI:** 10.1371/journal.pone.0277416

**Published:** 2022-11-16

**Authors:** Leela McKinnon, David R. Samson, Charles L. Nunn, Amanda Rowlands, Katrina G. Salvante, Pablo A. Nepomnaschy

**Affiliations:** 1 Department of Anthropology, University of Toronto Mississauga, Mississauga, Ontario, Canada; 2 Department of Evolutionary Anthropology, Duke University, Durham, NC, United States of America; 3 Duke Global Health Institute, Duke University, Durham, NC, United States of America; 4 Maternal and Child Health Laboratory, Faculty of Health Sciences, Simon Fraser University, Burnaby, British Columbia, Canada; The University of Sydney, AUSTRALIA

## Abstract

Sleep duration, quality, and rest-activity pattern—a measure for inferring circadian rhythm—are influenced by multiple factors including access to electricity. Recent findings suggest that the safety and comfort afforded by technology may improve sleep but negatively impact rest-activity stability. According to the circadian entrainment hypothesis, increased access to electric lighting should lead to weaker and less uniform circadian rhythms, measured by stability of rest-activity patterns. Here, we investigate sleep in a Maya community in Guatemala who are in a transitional stage of industrialization. We predicted that (i) sleep will be shorter and less efficient in this population than in industrial settings, and that (ii) rest-activity patterns will be weaker and less stable than in contexts with greater exposure to the natural environment and stronger and more stable than in settings more buffered by technologic infrastructure. Our results were mixed. Compared to more industrialized settings, in our study population sleep was 4.87% less efficient (78.39% vs 83.26%). We found no significant difference in sleep duration. Rest-activity patterns were more uniform and less variable than in industrial settings (interdaily stability = 0.58 vs 0.43; intradaily variability = 0.53 vs 0.60). Our results suggest that industrialization does not inherently reduce characteristics of sleep quality; instead, the safety and comfort afforded by technological development may improve sleep, and an intermediate degree of environmental exposure and technological buffering may support circadian rhythm strength and stability.

## Introduction

The association between rapid urbanization and sleep disruption is an emerging public health concern. Insufficient sleep and circadian rhythm dysregulation are associated with numerous physiological pathologies in metabolic, immune, and cardiovascular processes [1–3]. A zeitgeber is an external, rhythmically occurring natural phenomenon which acts as a time cue in the regulation of the body’s circadian rhythms. One of the most studied zeitgebers is light, but several other non-photic stimuli can interact with the body’s circadian pacemaker to entrain (i.e., regulate, synchronize) circadian rhythms. For example, ambient temperature, food availability, physical activity, and sociality are all non-photic stimuli that, at least in mammals, have a strong influence on the entrainment of circadian rhythms [4]. Electric lighting, which extends light exposure past natural sunset, and temperature-controlled housing, which allows maintenance of a constant temperature free of daily fluctuations, are recent technological developments in human evolutionary history. Until their emergence, sleep behavior was shaped by a complex interaction of physiological processes modulated by zeitgebers, including the natural light-dark cycle and environmental temperature fluctuations [5–8].

In humans, greater exposure to natural sunlight and less exposure to artificial lighting are associated with internal circadian clock synchronization such that bedtime and wake times are less variable and occur earlier, closer to sunset and sunrise [9]. Harmful effects of artificial lighting have been demonstrated in both industrial [10,11] and small-scale, non-industrial contexts [12,13], with the increased reports of sleep disturbance in metropolitan settings leading many to identify an emerging sleep loss epidemic [14,15]. In contrast, building evidence suggests that the comfort and safety of sleep environments that are buffered from noise, light, and ambient temperature fluctuation may facilitate longer, higher quality sleep [8,16]. These mixed results suggest that multiple factors may influence sleep, including natural light rhythms, subsistence activities, degree of industrialization, and cultural elements.

Understanding the synergistic effects of these factors requires studying populations that vary in their level of exposure to them. These studies should inform our understanding of sleep and human evolution and the mechanisms through which cultural change occurs, as well as informing sleep health research in the context of global technological advances and industrialization. To date, the majority of sleep data come from populations at the extreme ends of the technological development spectrum: nonindustrial communities considered to demonstrate “natural” human sleep, with continuous exposure to both natural light and ambient temperature variation, and industrial populations that are highly technologically buffered from these circadian regulators.

Here, we expand on the current literature of sleep and circadian rhythms in nonindustrial settings by investigating sleep and rest-activity patterns, measures used for inferring circadian rhythm [17], among the inhabitants of a small Mayan community in Guatemala. This community obtained access to electric service only a few decades ago and is still undergoing the technological transition. The transition process provides an opportunity to investigate the relative contribution of environmental, cultural, and technological variables as modulators of sleep patterns.

The effects of nighttime artificial lighting and technological development on sleep have been investigated in other small-scale subsistence societies [18]. Comparisons between small-scale subsistence groups who use electricity and those who rely only on natural light demonstrate the effects of artificial light on sleeping patterns. Use of electrically powered lights has been found to delay bedtimes in small-scale subsistence groups in Argentina, Mozambique, Vanuatu, and Brazil [19–21]. A consistent pattern is emerging: exposure to artificial nighttime lighting in small-scale subsistence populations proves to be associated with later sleep and wake time, and in some cases decreased sleep duration. Widespread use of artificial lighting appears to be associated with less circadian rhythm stability (the similarity of rest-activity on individual days) as well. The association between sleep and temperature has been demonstrated in Hadza hunter-gatherers, where homes have minimal buffering from the substantial drop in temperature overnight; warmer temperature is associated with longer sleep duration [22]. In addition, initiation of sleep periods in Hadza and Tsimane groups is associated with the period of falling temperature [8], further demonstrating the role of temperature as an important zeitgeber [23].

According to the circadian entrainment hypothesis, limited access to electric power and temperature regulating technology should lead to greater exposure to zeitgebers, which facilitates entrainment of circadian rhythms. Thus, circadian rhythms are expected to be more stable and uniform in resource-limited settings than in more developed settings where exposure to zeitgebers, such as the natural light-dark cycle, are buffered [16]. For example, circadian rhythms in Malagasy small-scale agriculturalist and Hadza hunter-gatherer communities with no access to electricity are of higher amplitude and stability than those in developed economies, while sleep is relatively short and of low quality [16,22].

In addition to cues in the physical environment, sleep patterns may be influenced by sex and gender, age, and social activity patterns. In industrialized contexts with gender egalitarian social norms, women’s sleep quality as determined by efficiency (percentage of time spent asleep out of the total amount of time in bed) tends to be higher and sleep duration longer, primarily based on studies from North America, Europe, and Australia [24,25]. Men consistently exhibit later chronotype (chronotype is the propensity to fall asleep and wake up earlier or later, often expressed as eveningness vs morningness) than women in industrial contexts, especially up to age 40, which has been attributed to a possible reproductive or social advantage to activity later in the evening [26–28]. However, no significant gender differences in sleep architecture (i.e., relative time spent in each sleep stage) as measured by electroencephalography have been reported [29].

To date, the circadian entrainment hypothesis has only been tested in populations with either high access or little access to artificial lighting and environmental temperature regulating technology [16], and the majority of studies reporting gendered sleep differences come from industrial settings. To investigate whether rapidly increasing exposure to electric lighting and technology affects sleep duration, efficiency, and circadian rhythm strength and stability, we compare these outcomes in the Maya community with those from a population of small-scale agriculturalist Malagasy [16], who have no electric infrastructure, and an industrial population from the United States. We also explore the effects of gendered labor roles and social norms to test hypotheses related to the effects of social factors as drivers of sleep and rest-activity patterns. Specifically, we tested the following hypotheses:

1. The circadian entrainment hypothesis [16]: greater entrainment of circadian rhythms should be facilitated by more exposure to zeitgebers and limited exposure to electricity and temperature regulating technology. In terms of industrialization and zeitgeber exposure, the Maya study population represents an “in between society.” The houses in the community have an electric grid that reaches most households. Electric service, however, is frequently interrupted. Ambient temperature buffering is limited and achieved via traditional architectural designs (e.g., adobe or concrete blocks as building material and uninsulated windows) and no electrical means to control it, such as air conditioners. Houses are mostly built with adobe or concrete blocks which protect inhabitants from the weather and partially from natural lights and moderately buffers them from temperature extremes. We predict that this moderate degree of buffering should allow for longer sleep duration and greater efficiency than in communities with more rustic housing and higher exposure to natural environmental stimuli. On the other hand, we predict that people in our study population should experience shorter sleep duration and lower sleep efficiency than those living in industrialized contexts with more artificial stimuli, where continuous use of heating and air conditioning maintains temperature and comfort. We expect rest-activity rhythms in our study population to be weaker and less stable than those of small-scale subsistence societies with more natural environmental (e.g., zeitgeber) exposure and stronger and more stable than those with more environmentally buffered sleep sites with lower exposure to natural light-dark cycles and temperature fluctuations.
2. The gendered sleep hypothesis: gender influences sleep patterns through gendered labor and social roles that contribute to men spending more time away from the house working outside, coupled with nighttime socialization. In this indigenous community, men are perceived to engage in nocturnal activities more frequently and to stay engaged in them until later than women. Thus, we predicted that gender would be associated with circadian entrainment, with men exhibiting shorter sleep duration, lower sleep efficiency, later central phase measure (i.e., the midpoint of the sleep period, indicating later chronotype), and more variable sleep and wake timing than women. Yet, men should experience lower fragmentation, greater stability, and higher amplitude (i.e., strength) of their rest-activity patterns due to more time working outside, and therefore exposure to environmental circadian regulators (e.g., sunlight). Alternatively, if only ecological drivers that entrain circadian rhythms (i.e., timing of sunset and sunrise) and meteorological variables (i.e., ambient temperature, humidity, and rainfall) drive sleep duration, efficiency, timing, and circadian rhythm strength and stability, then we should see no gender differences in sleep or rest-activity patterns. Both sex and gender can affect sleep patterns, and these two effects are difficult to disentangle. We use the term gender to best capture the effects of social expectations and gender roles in shaping sleep and activity patterns.

## Methods

### Study location and participants

**Fig 1:**
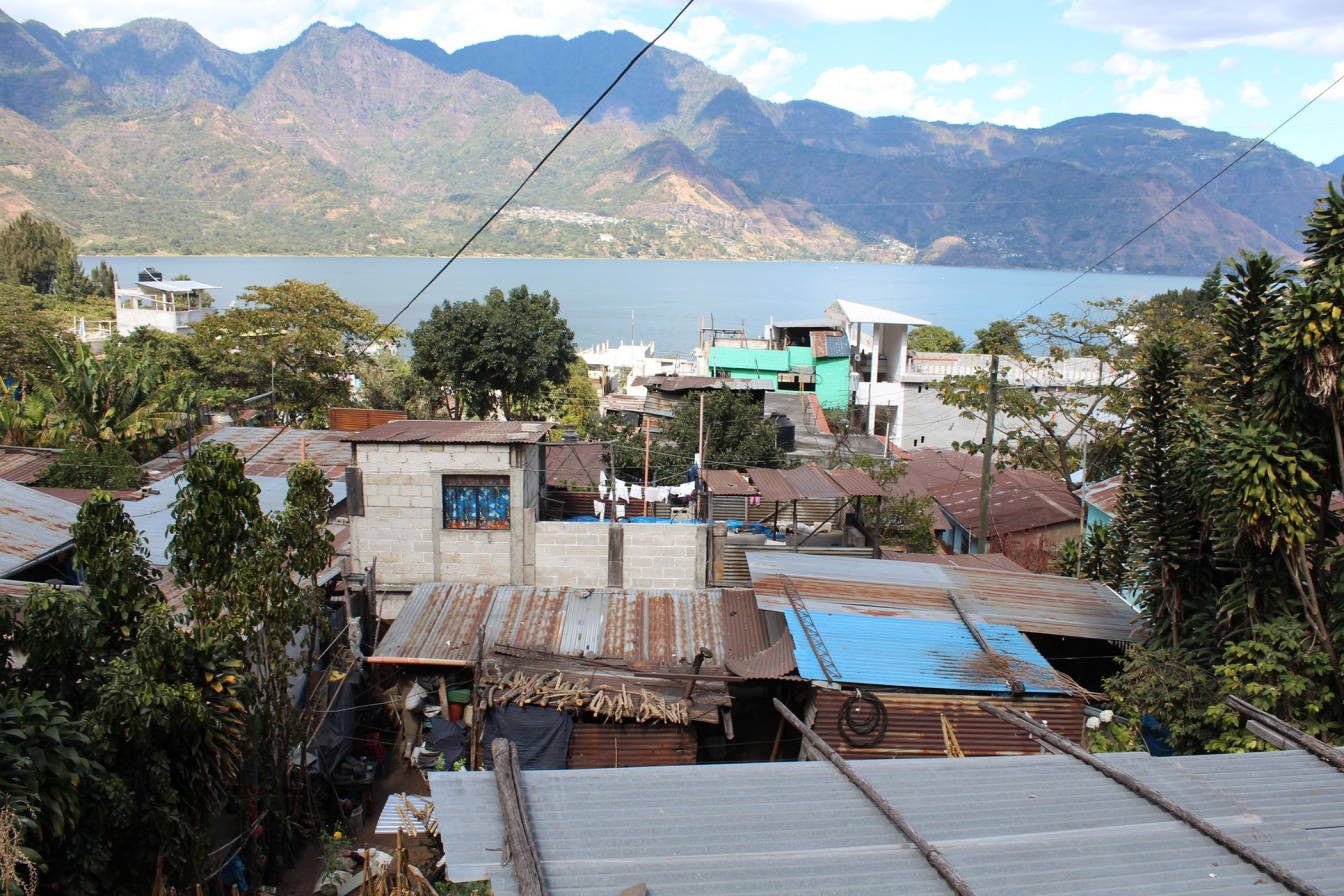
Houses in the Kaqchikel Maya community. The tin roofs that cover most houses are visible, and both adobe and concrete building material can be seen.

Members from a Kaqchikel Maya community living in a small agricultural town in the highlands of western Guatemala were recruited to participate in this study. Houses in this town are built close to each other around a main square that includes the municipal building, a health post, a school and a *pila* (a communal water source and water basins for clothes washing). The town is surrounded by agricultural land, as small-scale agriculture was the main economic activity until recently. Most houses in the community are built with adobe or concrete blocks and have tin roofs, with windows covered by wood panels or curtains (Fig 1). In general, houses do not include technological means for temperature regulation; hence, this population tends to be more exposed to meteorological changes than those in urban contexts with access to air conditioning or central heating. This community is undergoing an economic and technological transition, but access to electricity is not constant and powered lighting or appliances such as televisions vary among households.

Many families are still involved in subsistence-level economic activities including farming and fishing. However, as the transition to a market economy advances and subsistence pattern shifts, both men and women are now increasingly working for comparatively wealthier national and international chalet owners that populate the Lake Atitlán region, now a popular tourist destination. As a result, social and economic stratification is advancing. Traditional agriculture has in many cases given way to weekend agriculture as a source of supplemental income. Most economic activities (agriculture, fishing, commerce, building and services) are manual and, like most social activities, diurnal. That said, men do spend time out with friends in the evenings and there are church activities and community committees that take place after work. On weekends, people go to the market, visit with family, and devote time to religious activities. Over the last two decades, men’s agricultural roles have declined as some have found jobs in construction and house maintenance for chalet owners. Women are also increasingly working outside the home as house cleaners, babysitters, hotel workers, and makers and sellers of handicrafts such as weaving, jewelry, and beadwork to visitors and re-sellers. In addition to following traditional divisions of labor by gender, traditional gender differences are pronounced in social activities as well, with men often devoting more time than women to nighttime socialization starting in late adolescence.

Data were collected from January to April 2017 for a total of 464 nights of data from 37 participants, 14 men (mean age = 30.71 years, range = 18–45 years, nights of data = 85) and 23 women (mean age = 45.76 years, range = 34.4–62 years, nights of data = 379). Nine men (mean age = 32.44 years, range = 23–45 years) and 20 women (mean age = 46.62 years, range = 34.4–62 years) had at least seven days of complete 24-hour data, and were thus included in Nonparametric Circadian Rhythm Analysis (NPCRA).

### Informed consent

Informed consent was obtained from all participants prior to the start of data collection. Participants were informed about the study protocols and goals in their native Kaqchikel language by Kaqchikel-speaking field assistants. They were informed that participation in the study was voluntary, and that they could withdraw from the study at any time. Those who agreed to participate indicated their consent with an X, thumbprint, initial, or signature (personal preference). Study protocol followed Simon Fraser University Research Ethics Board, SFU-REB protocol 2016s0577 and the University of Toronto, Mississauga Research Ethics Board, UTM-REB protocol 35696.

### Equipment

Sleep-wake data were obtained with the CamNtech Motionwatch 8, a wrist actigraphic device that provides measures of sleep and wake activity. Polysomnography, which measures brain activity to distinguish sleep and wake activity as well as sleep stage, is still considered the gold standard in sleep studies in terms of reliability and accuracy. However, polysomnography often necessitates laboratory-based study, with invasive equipment attached to the participant. Actigraphs are noninvasive, and do not interfere with daily activity. Using a built-in accelerometer, actigraphs quantify movement in one-minute epochs; these data are then algorithmically translated to a binary sleep-wake determination. Using the high-sensitivity setting to determine sleep, our wake threshold was defined as a period of 20 consecutive bouts that were categorized as wakefulness. To assist in actigraphy scoring accuracy, participants were asked to press an event marker button upon going to bed and getting up, which increased reliability in determining sleep onset and offset. Actigraphs are validated against polysomnography at 96.5% sensitivity and 86.3% accuracy [30]. Specific validation of the CamNtech Motionwatch 8 demonstrates its reliability in estimating sleep parameters including sleep duration and efficiency [31].

### Statistical analysis and hypothesis testing

Raw actigraphy data were processed and compiled using CamNtech Motionware analysis software, and analyzed using R Core Team [32]. To test **the circadian entrainment hypothesis**, we used NPCRA, which is a method of assessing the day-night rhythm in activity level [33] and is used to generate individual values for relative amplitude (RA; a measure expressing the ratio of inactivity during sleep periods and activity during wake periods, expressed as a range of values between 0 and 1 with higher values indicating higher rhythm amplitude), interdaily stability (IS; the similarity of activity patterns on different days, expressed as a range of values between 0 and 1 with 0 indicating a complete lack of rhythm and 1 indicating complete stability of rhythm), and intradaily variability (IV; a measure of fragmentation of rest and activity periods, expressed as a value between 0 and 2 with higher values indicating more variability in rhythm) [34]. Calculation of RA, IS, and IV provide a quantitative description of the amplitude (i.e., strength), stability, and variability of the rest-activity rhythm [33]. These non-parametric variables infer key characteristics of circadian rhythm, including synchronization with a zeitgeber [17]. Additionally, these non-parametric rest-activity variables have been used previously to infer and describe circadian rhythms in nonindustrial populations [16,22], as well as in clinical assessment of circadian function [35]. It is recommended that a minimum of 7 days of complete 24-hour actigraphy data be used to compute NPCRA [34]; we therefore focused on the 29 Maya participants who satisfied this requirement.

Using unpaired, two-sample Student’s t-tests or Mann-Whitney U tests, we compared the Maya participants’ sleep and rest-activity rhythm averages with those from participants in Madagascar and the United States. Comparison between Malagasy participants and industrial populations have been previously reported by Samson and colleagues [16]. Malagasy participants are small-scale, rural agriculturalists who live in a community in northeastern Madagascar. This community has no electric infrastructure, and houses are exposed to environmental temperature fluctuation and nighttime noise. Therefore, Malagasy sleep is considered representative of a group living with minimal exposure to the effects of electricity and in-home temperature and noise buffering. We used comparative sleep data from a study conducted in July and August 2015 and 2016 from 21 Malagasy individuals (female mean age = 40.30 years; male mean age = 36.80 years; age range = 19–59 years), published by Samson and colleagues [16]. Written informed consent was obtained from participants following the protocol provided by the Duke University Institutional Review Board for human subjects research. All participants were informed of the study objectives and procedures. Upon obtaining written consent, they were instructed to wear the watch continuously for the duration of the study period and asked to use the event marker button to indicate when they went to bed and got up. Movement was measured in 60-second intervals and scored using CamNtech software [16].

For comparative data from individuals living with continuous, 24-hour access to electricity characteristic of industrial populations, we used data from the Midlife in the United States (MIDUS) cohort. The MIDUS project recruited participants from throughout the United States, who were interviewed by phone and self-administered questionnaire, answering questions related to demographic variables, health, employment, and psychological factors [36]. Actigraphy data are available in the MIDUS II and MIDUS Refresher datasets. MIDUS II was conducted in 2004 and included follow-up of the data collected in MIDUS I as well as cognitive, neurological, and comprehensive biomarker assessments, and sleep data from subsamples of respondents [37]. MIDUS Refresher data were collected between 2012 and 2016 and include comprehensive biological assessments of 3,577 respondents [38]. Actigraphy data were generated with the Mini Mitter Actiwatch-64 (Philips Healthcare, Amsterdam, Netherlands) activity monitor. Participants were instructed to wear the watches continuously for seven consecutive days, with movement collected in 30-second intervals. Data were scored and analyzed using Aciwatch software [39]. Protocol for data collection and written informed consent was approved by the Education and Social/Behavioral Sciences and the Health Sciences Institutional Review Boards at the University of Wisconsin-Madison.

We selected MIDUS participants with ages between 25 and 62 to match the Maya sample, and excluded participants who reported sleep disorders and those taking sleep medication more than once per week. After excluding based on these criteria, 289 individuals (mean age = 47.07 years; age range = 25–62) were included for sleep comparisons, with 276 (mean age = 47.08 years; age range = 25–62) of these having circadian rhythm data for rest-activity comparisons.

To date, relatively few studies have reported population-wide sleep characteristics that include both sleep and rest-activity rhythm quotas; therefore, we chose these three populations as example groups to better contextualize Maya sleep and rest-activity rhythms. Once these data become available, future work should perform formal comparative analysis across cultures (especially subsistence strategies). Although it is possible that the chosen populations are not representative of their general demographic and geographic characteristics, they still reveal the capacity of the population in question [40].

To test **the gendered sleep hypothesis**, we used the *lme4* package in R to model data using linear mixed effects models, controlling for temperature, rainfall, and humidity. We included temperature in our models because of the reported associations between temperature and sleep in small-scale subsistence groups [8,22]. While rainfall and humidity are less well reported in the sleep literature, these variables have the potential to influence comfort during the sleep period and thus to affect sleep patterns, particularly in sleep spaces where there is minimal buffering from changing environmental conditions [23,34].

Temperature, humidity, and rainfall measures were obtained using Historical Forecast Weather (HFW) data downloaded from World Weather Online (https://www.worldweatheronline.com/hwd/hfw.aspx). HFW data are generated using raw data from world weather agencies and then applying factors such as terrain and altitude. HFW provides hourly weather forecasts, and distinguishes between daytime and nighttime readings. After excluding daytime measures, we calculated nightly averages for temperature and humidity and nightly cumulative rainfall using hourly HFW readings between 19:00 (7:00 pm) on the day of interest and ending the following day at 5:00 (5:00 am), which are the hours categorized as night by World Weather Online.

We built three separate linear mixed effects models to investigate the predictors of sleep duration, sleep efficiency, and central phase measure (e.g., Sleep duration ~ Age*Gender + Rainfall + Humidity + Temperature + 1|subject), with subject as a random effect for individual participant to control for repeated measures [34]. Male was set as the reference gender for all models. **Sleep duration**, or total sleep time, is the amount of time scored as sleep on actigraphic analysis. Statistical analysis and results are reported as total sleep time in hours. **Sleep efficiency** is the percentage of time spent asleep out of the total time in bed in the period scored as sleep on actigraphic analysis and is one of the quantitative measures that contributes to sleep quality [34]. **Central phase measure** indicates the midpoint of the sleep period as a minute value before or after midnight and is used to assess circadian timing of sleep [34]. Values can be positive or negative, with higher values indicating a later sleep midpoint and a more evening-preferring chronotype, and lower values indicating an earlier sleep midpoint and greater morning preference. Models were averaged using the *MuMIn* package in R, which pools information from more certain estimates in the model to improve less certain estimates [41]. Along with estimate, standard error, and confidence interval, we report the importance of each predictor in our averaged model. Importance is reported in a range of 0 to 1.

Sleep timing consistency was compared by measuring variance in consistency of sleep onset (i.e., falling asleep) and offset (i.e., waking up), using an F-test of variance between men and women. Men’s and women’s sleep averages were compared with the *BayesFactor* package in R, using default priors [42]. This Bayesian approach (e.g., ttestBF(sleep parameter ~gender + ID) allowed us to control for repeated measures by ID and to compare the probability of the alternative to the null hypothesis (no effect). Bayes factor results indicate the likelihood of the data occurring if the null hypothesis is true compared to the alternative hypothesis being true. Negative Bayes factor values that are further from zero provide stronger evidence for the null hypothesis, and positive values further from zero indicate stronger support for the alternative hypothesis. Interpretation for Bayes factor values is adapted from Jarosz and Wiley [43].

We report additional sleep variables that were generated with CamNtech Motionware analysis software. These include sleep latency (time in hours between lights out and sleep onset), wake after sleep onset (WASO; periods of awakening during the sleep period, expressed in hours), and sleep fragmentation (a quantitative index of restlessness in the sleep period, calculated as the sum of the percentage of sleep epochs spent moving and the percentage of immobile periods during the night).

## Results

Comparisons of sleep characteristics of the Maya sample to the Malagasy and United States samples are presented in Table 1. Malagasy participants (9.4 hours) spent significantly longer time in bed compared to Maya participants (8.33 hours; t = -2.66; p = 0.012). At 6.52 hours, sleep duration in the Maya participants is about the same as in the Malagasy participants (6.5 hours), but significantly longer than in the United States (6.38 hours, t = 2.27, p = 0.024). Maya participants’ sleep efficiency of 78.39% is significantly greater than Malagasy participants’ sleep efficiency (70.70%; t = 2.54, p = 0.016) but lower than the United States sample (83.26%; W = 289183, p < 0.001). WASO in the Maya participants (1.51 hours) is less than the Malagasy participants (2.10 hours; t = -2.60; p = 0.014) and greater than the United States sample (0.69 hours; W = 819332; p < 0.001). Sleep fragmentation index is lower in the Maya sample (31.91) compared to the Malagasy sample (45.90; t = -3.34; p = 0.002).

**Table 1:** Sleep quotas comparing the full Maya sample with Malagasy participants [16] and an industrial sample from the United States.

Sleep quota	Maya	Malagasy	United States
N	37	21	289
Age (years)	40.07	36.80 (men); 40.30 (women)	47.07
Gender: Woman (%)	23 (62.16%)	9 (42.86%)	160 (55.36%)
Gender: Man (%)	14 (37.84%)	12 (57.14%)	129 (44.64%)
Sleep onset	22:07 (1:05)	19:21 (3:38)	23:25 (1:21)
Sleep offset	6:09 (1:05)	05:44 (0:53)	06:35 (1:24)
Time in bed (hours)	8.33 (1.22)	9.40 (1.60)*	7.07 (1.53)***
Sleep latency (hours)	0.21 (0.33)	0.55 (1.15)	0.37 (0.56)***
Sleep duration (hours)	6.52 (1.14)	6.50 (1.60)	6.38 (1.40)*
Wake after sleep onset (hours)	1.51 (0.69)	2.10 (0.90)*	0.69 (0.45)***
Sleep efficiency (%)	78.39 (8.75)	70.70 (12.20)*	83.26 (9.93)***
Sleep fragmentation	31.91 (13.20)	45.90 (16.40)**	Not reported

Values are reported as mean (standard deviation), and significance is denoted with stars relative to the Maya sample. Significance codes:

* p < 0.05

** p < 0.01

*** p < 0.001.

Table 2 shows a comparison of rest-activity patterns between Maya, Malagasy, and United States participants. Maya participants have significantly higher interdaily stability (0.58; W = 6111, p < 0.001) than the United States sample (0.43), and lower intradaily variability (0.53) than both Malagasy (0.67; t = -2.23, p = 0.038) and United States (0.60; W = 3080, p = 0.041) participants. There is no difference in relative amplitude between the three samples.

**Table 2:** NPCRA comparing Maya, Malagasy [16], and industrial United States participants.

Parameter	Maya	Malagasy	United States
N	29	10	276
Age (years)	42.22 (10.60)	Range = 19–59 years	47.08 (9.01)
Gender: Woman (%)	20 (68.97%)	5 (50.00%)	151 (54.71%)
Gender: Man (%)	9 (31.03%)	5 (50.00%)	125 (45.29%)
Interdaily stability	0.58 (0.28)	0.51 (0.08)	0.43 (0.09)***
Intradaily variability	0.53 (0.19)	0.67 (0.16)*	0.60 (0.13)*
Relative amplitude	0.92 (0.07)	0.91 (0.05)	0.92 (0.06)

Participants included in NPCRA comparisons had at least seven days of complete actigraphy data. Values are reported as mean (standard deviation), and significance is denoted with stars relative to the Maya sample. Significance codes:

* p < 0.05

** p < 0.01

*** p < 0.001.

We further controlled for age and gender in supplementary analyses using linear mixed effects models for sleep variables and linear regression models for rest-activity variables between the Maya and MIDUS samples. After including age and gender as covariates in our linear mixed effects model, cohort remains a significant predictor of time in bed (estimate = -0.26, SE = 0.04, p < 0.001), sleep latency (estimate = 0.12, SE = 0.04, p = 0.003), sleep efficiency (estimate = 0.19, SE = 0.05, p < 0.001), and WASO (estimate = -0.51, SE = 0.04, p < 0.001). However, after controlling for age and gender, differences between Maya and MIDUS sleep duration are no longer significant (estimate = 0.001, SE = 0.05, p = 0.986). Linear regression results of rest-activity variables show that Maya cohort membership still predicts greater interdaily stability (estimate = -0.15, SE = 0.02, p < 0.001) and intradaily variability (estimate = 0.07, SE = 0.03, p = 0.015) after controlling for age and gender. Full results of supplementary linear mixed effects models are presented in S1 Table and supplementary linear regression models are presented in S2 Table.

**Fig 2:**
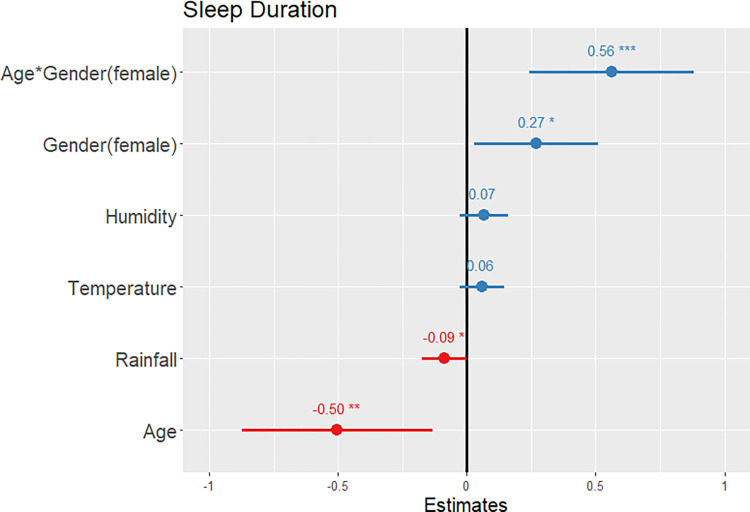
A prediction plot of standardized fixed effects for sleep duration. Longer sleep duration is predicted by the interaction of age and gender, gender, and negatively predicted by age and rainfall. Significance codes: * p < 0.05; ** p < 0.01; *** p < 0.001.

**Fig 3:**
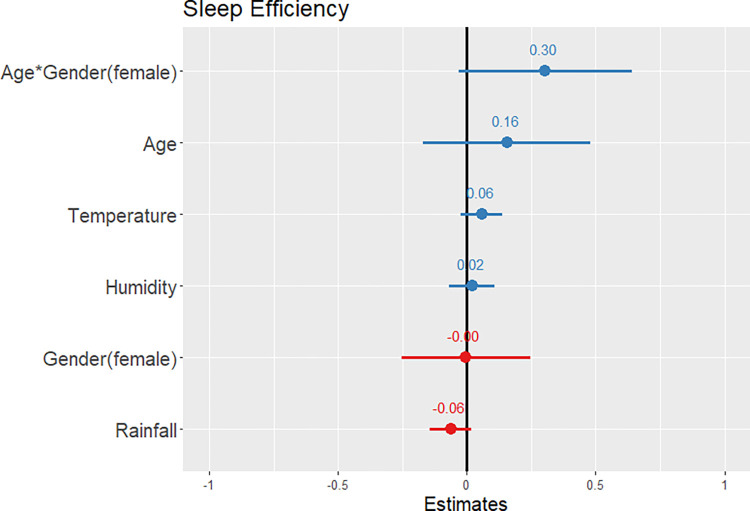
A prediction plot of standardized fixed effects for sleep efficiency. None of the included variables were significantly associated with sleep efficiency. Significance codes: * p < 0.05; ** p < 0.01; *** p < 0.001.

Descriptive statistics and statistical test results for sleep differences between Maya men and women are presented in Table 3, and rest-activity differences are presented in Table 4. The linear mixed-effects model showed that sleep duration (S3 Table) significantly increased with an interaction of age and gender (β ± SE = 0.56 ± 0.16, p < 0.001, confidence interval [CI] = 0.24 to 0.88) and gender (female) (β ± SE = 0.27 ± 0.12, p = 0.027, CI = 0.03 to 0.51), but decreased with age (β ± SE = -0.50 ± 0.19, p = 0.008, CI = -0.87 to -0.13) and rainfall (β ± SE = -0.09 ± 0.04, p = 0.046, CI = -0.17 to -0.002; see Fig 2). In our efficiency model (S4 Table), sleep efficiency was not significantly associated with gender (β ± SE = -0.003 ± 0.13, p = 0.978, CI = -0.25 to 0.25) or any other variables included in the model (Fig 3).

**Table 3:** Descriptive statistics: Mean (SD) and statistical tests for gender differences in sleep quotas.

	Men	Women	Bayes Factor	Interpretation
N	14 (37.84%)	23 (62.16%)		
Age (years)	30.71	45.76		
Sleep onset	23:07 (1:17)	21:54 (0:54)		
Sleep offset	6:59 (1:42)	5:58 (0:46)		
Time in bed (hrs)	8.14 (1.78)	8.38 (1.05)	0.44	Weak evidence
Sleep latency (hrs)	0.19 (0.32)	0.22 (0.34)	0.15	Substantial evidence for null hypothesis
Sleep duration (hrs)	6.24 (1.46)	6.58 (1.05)	2.39	Weak evidence
Wake after sleep onset (hrs)	1.63 (0.65)	1.48 (0.70)	0.63	Weak evidence
Sleep efficiency (%)	76.83 (7.57)	78.74 (8.97)	0.64	Weak evidence
Sleep fragmentation	35.83 (12.31)	31.04 (13.25)	10.76	Strong evidence for alternative hypothesis
Central phase measure	183.48 (75.01)	116.17 (39.58)	2.97	Weak evidence

Subjects are men and women age 18–62 years.

**Table 4:** Descriptive statistics: Mean (SD) and statistical tests for gender differences in circadian rhythm.

	Men	Women	Bayes Factor	Interpretation
N	9 (31.03%)	20 (68.97%)		
Age (years)	32.44	46.62		
Relative amplitude	0.89 (0.12)	0.94 (0.04)	1.37	Weak evidence
Interdaily stability	0.65 (0.45)	0.56 (0.17)	0.47	Weak evidence
Intradaily variability	0.49 (0.10)	0.55 (0.22)	0.46	Weak evidence
L5	1710 (2105)	817 (416)	1.26	Weak evidence
M10	26161 (4928)	28728 (8765)	0.47	Weak evidence
L5 onset	10:46	11:26		
M10 onset	8:33	7:42		

Subjects are men and women age 23–62 years.

**Fig 4:**
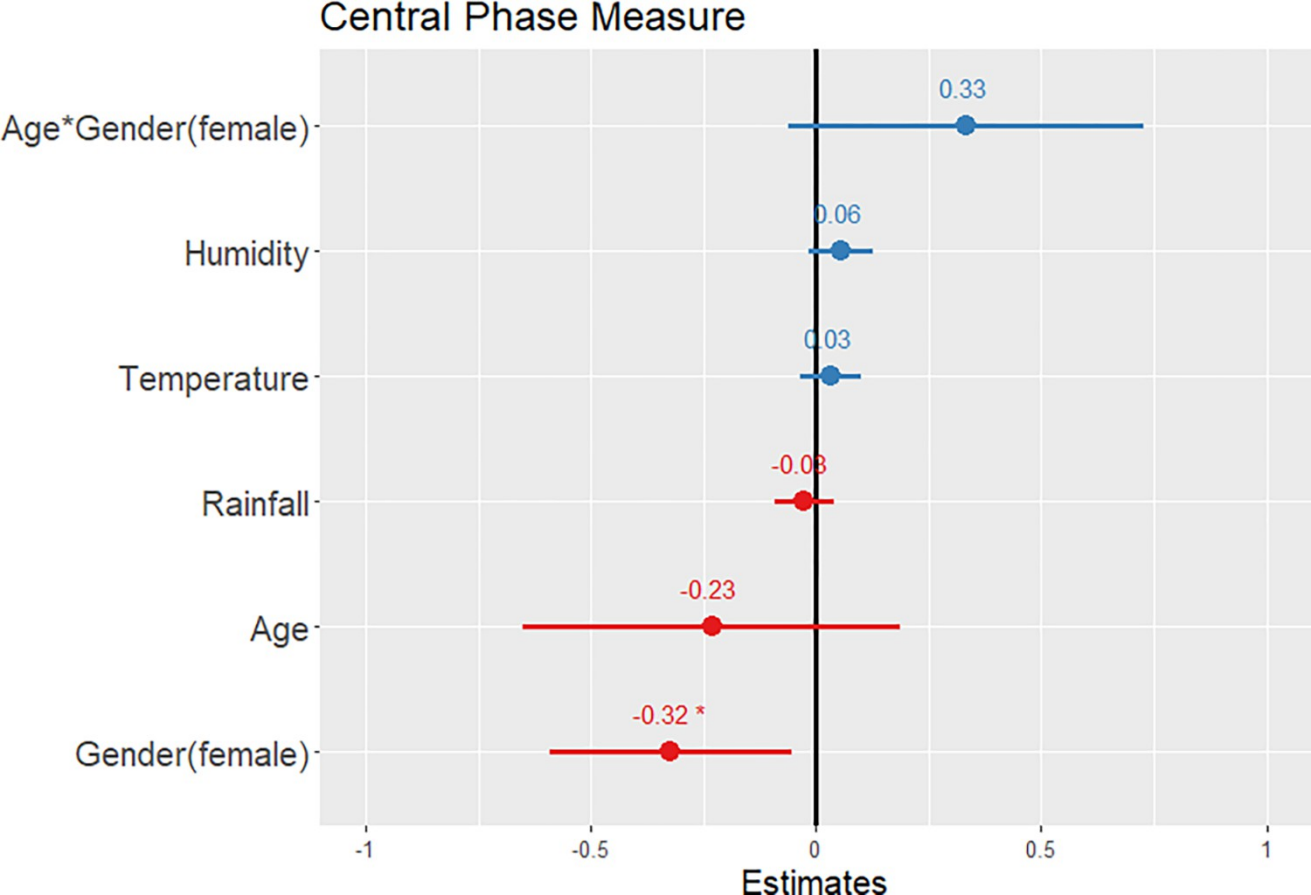
A prediction plot of standardized fixed effects for central phase measure. Earlier central phase measure is predicted by gender. Significance codes: * p < 0.05; ** p < 0.01; *** p < 0.001.

**Fig 5:**
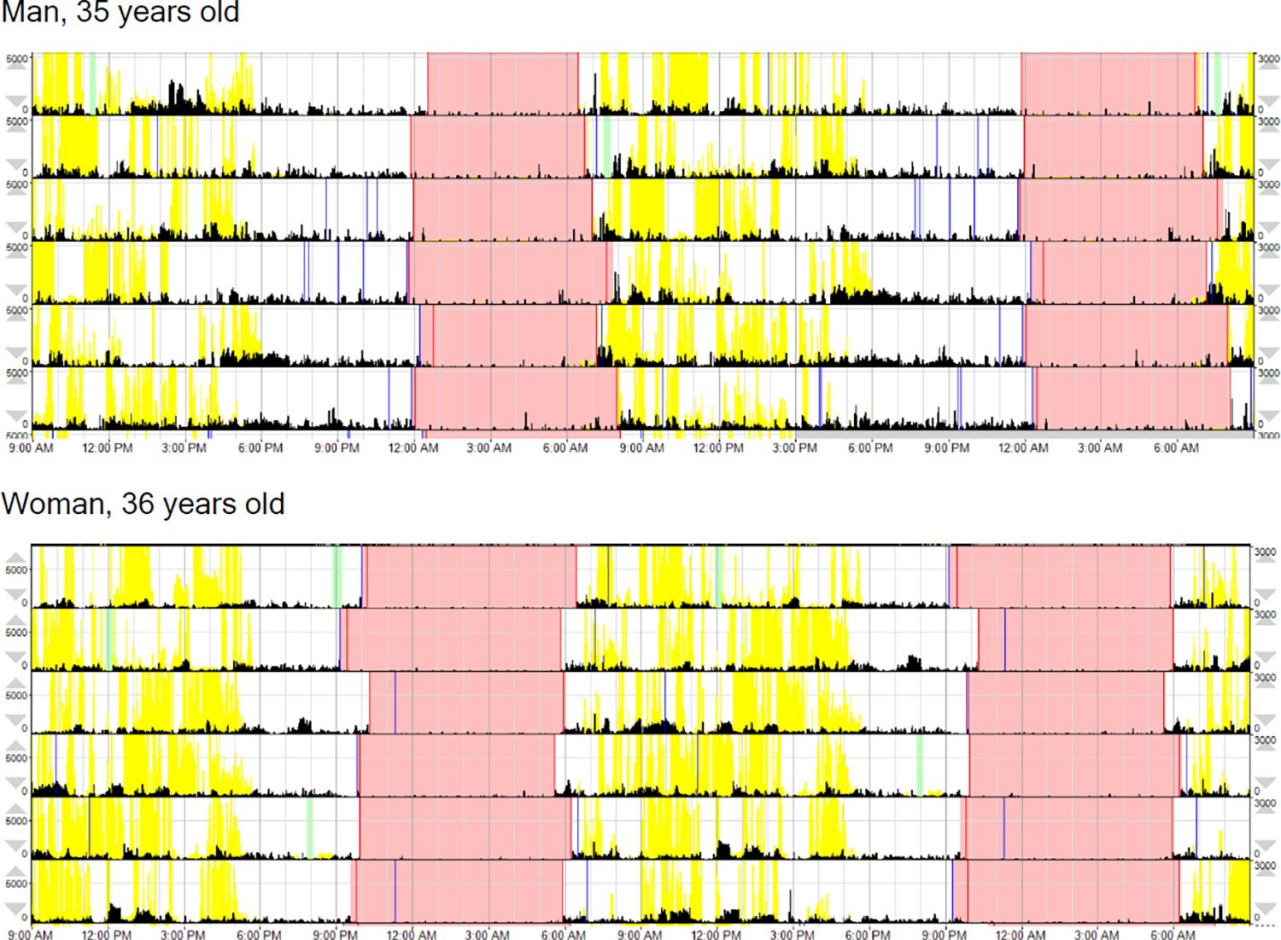
Actograms for a 35-year-old Maya man and a 36-year-old Maya woman. The red-shaded portions indicate sleep periods. Activity counts are shown by the black bars, and light exposure is represented by yellow. The absence of light during the night in these graphs is representative of the sample, and illustrates the light-blocking advantages of structural buffering. The graphs of men’s and women’s sleep patterns visualize the later bedtimes and waketimes of men compared to women, as well as the greater variance in sleep offset (i.e., wake) timing.

Results of our third model (S5 Table) showed that central phase measure was lower in women (β ± SE = -0.32 ± 0.14, p = 0.018, CI = -0.59 to -0.06; see Fig 4), indicating significantly earlier (i.e., more morning-preferring) chronotype. The later midpoint of men’s sleep compared to women’s is visualized in the example actogram (graph of sleep and wake activity) in Fig 5. Consistent with predictions under the gendered sleep hypothesis, we found significantly greater variance for men’s sleep onset and offset compared to women (sleep onset: male mean = 23:07, variance = 0:04; female mean = 21:54, variance = 0:02; p < 0.001; sleep offset: male mean = 6:59, variance = 0:07; female mean = 5:58, variance = 0:01; p < 0.001).

## Discussion

### The circadian entrainment hypothesis

Our results are consistent with the circadian entrainment hypothesis. Sleep was less efficient among Maya participants than among those in more industrialized settings. Maya participant interdaily stability, a quantitative measure of regularity in the rest-activity pattern, was greater compared to the industrial United States sample, and persisted after controlling for age and gender. Placed within the broader context of sleep in other nonindustrial populations, sleep in the Maya sample was longer and more efficient than sleep in Hadza hunter-gatherers (6.25 hours, 68.9% efficiency) [22] and Himba pastoralists (5.47 hours, 66.4% efficiency) [44]. However, Maya participant sleep duration was about the same as that reported in Tsimane forager-horticulturalists (6.63 hours) [45], and shorter and of lower efficiency than rural Haitians (7.0 hours, 88.7% efficiency) [46] and industrialized populations in Italy (7.60 hours, 94.2% efficiency) [47] and the United States (7.02 hours, 89.9% efficiency) [48].

The sleep-delaying effects of artificial lighting are evident in a number of comparative studies. For example, greater exposure to outdoor nighttime lighting in urban United States settings is associated with later sleep and wake time, shorter sleep duration, and increased daytime sleepiness than in rural ones [11]. The use of light-emitting E-readers at night has been shown to result in longer time to fall asleep and reduced daytime alertness, fueling the concern that round the clock access to indoor lighting and use of light-emitting technologies are delaying bedtimes, leading to insufficient sleep duration [10]. In a comparative study between two small-scale subsistence groups in Argentina, exposure to natural light only was linked to longer average sleep duration than when there was exposure to electric infrastructure. This decrease in nightly sleep duration in those using electricity was associated with delayed bedtimes [12]. Similarly, individuals with access to electricity in rubber plantations in the Amazon rainforest experienced later bedtime and shorter sleep duration on workdays than those without access [13]. Participants who used artificial lighting exhibited delayed melatonin onset, demonstrating the potential physiological effect of nighttime exposure to light. In contrast, Hadza and Himba populations experience comparatively shorter, lower quality sleep despite lack of electric infrastructure. Similarly, our results of moderately long sleep duration and efficiency from the Maya community suggest that a presence of technology is not always associated with shorter, poorer quality sleep. Therefore, our findings add to a growing understanding that the relationship between industrialization and sleep is not linear, and is rather shaped by a complex interaction of environmental and social factors.

In our linear mixed effects models of sleep duration, efficiency, and central phase within the Maya sample, we found that rainfall was significantly associated with shorter sleep duration. It is possible that the tin roofs that are present on many of the houses in the sample community lead to noise from rainfall that delays or interrupts sleep. Our data were collected during January, a relatively dry part of the year. A comparison with data collected during the rainy season and inclusion of noise data in our models would help to clarify the association between rainfall and sleep duration. Neither temperature nor humidity had a significant association with sleep variables in our sample. While temperature in particular has been previously associated with sleep duration and timing, it is possible that the houses of the Maya participants—which are often made of adobe, a good temperature and humidity buffer—are more protected from the elements than typical housing in the small-scale subsistence groups discussed (e.g., Hadza), and may offer sufficient environmental protection to protect sleep from substantial disturbance due to temperature extremes or high humidity.

Our results add to previous findings pointing to the fortifying effect of environmental exposure on circadian rhythms. Using rest-activity patterns to infer characteristics of circadian rhythm, we found that relative amplitude (rest-activity rhythm strength) of 0.92, interdaily stability (rest-activity rhythm stability) of 0.58, and intradaily variability (rest-activity rhythm fragmentation) of 0.53 indicate that, in general, Maya participants’ rest-activity pattern robusticity was strong. This rest-activity rhythm robusticity was apparent when the Maya sample’s results were compared to an industrial population from the United States. Maya participants had more stable rhythms (interdaily stability) and less fragmented rhythms (intradaily variability) than their United States counterparts, although we did not find any difference in the expression of rest-activity amplitude.

Interestingly, Mayan participants did not appear to differ from their Malagasy counterparts in rest-activity rhythm stability, had marginally less rest-activity fragmentation, and showed no significant difference in the expression of relative rest-activity amplitude. In the Maya participants, exposure to nighttime lighting was minimal during the study period, illustrated in the representative actograms in Fig 5. These findings suggest that having more environmentally-buffered sleep sites, with reduced exposure to natural light and temperature fluctuations, combined with individual exposure to the natural environment, daily outdoor activities, and/or social regulation of sleep-wake timing may also influence circadian rhythm expression. If natural sunlight and limited use of artificial lighting are important for synchronization between environmental cues and physiology [9], it follows that urbanization and technological development could explain the increase in reports of sleep disturbance in metropolitan populations [49,50]. This increase may be due to circadian disruption. Overall, our results emphasize that sleep patterns’ complexity cannot be fully explained by population industrialization, and provide further support for the beneficial effects of some technological buffering from environmental exposure.

### The gendered sleep hypothesis

Our test of the gendered sleep hypothesis yielded mixed results. Consistent with our predictions, we found that women’s sleep was longer than men’s, and that men had significantly later chronotype (later bedtime and wake times). Furthermore, men showed significantly more variable sleep onset and offset, suggesting that work and social activity patterns differ between men and women. These results are consistent with our personal observations of the daily work and social schedules in this community. Women are responsible for making breakfast for the household, which involves early morning trips to the mill to obtain corn for tortillas. Men tend to socialize more at night, which could plausibly drive later bedtimes. Alternatively, we considered that ecological drivers (i.e., timing of sunset and sunrise that entrain circadian rhythms) and meteorological variables (i.e., temperature, humidity, and rainfall) that are uniform across the population may be a more important driver of sleep patterns, in which case we predicted no sleep differences by gender. While we observed significant differences between men and women in sleep duration and timing, no significant gender differences were found in rest-activity rhythm variables. Therefore, it appears that socio-ecological influences shared by both men and women in this population are important and that they may be contributing to circadian rhythm regulation.

Our findings in tests of the gendered sleep hypothesis are consistent with other findings in similar cultural contexts. Recent results from nonindustrial settings point to the importance of cultural factors in shaping gender differences in sleep. Prall and colleagues [44], for example, report extremely short sleep in Himba pastoralists of Namibia, who have been recorded to sleep just 5.47 hours per night on average [44], in stark contrast to sleep duration averages in industrialized populations, which tend to fall within the 7–8 hour range (e.g., [47,48]). Short sleep durations are especially pronounced in Himba men, with women presenting longer sleep duration (5.92 hours) and higher efficiency (70.2%) on average than men (average duration = 4.76 hours, average efficiency = 60.3%). The authors posit that high levels of young male social activity combined with daily labor demands of pastoralism are likely responsible for the dramatically low average sleep duration exhibited by men. A wide age range (15–78 years) of Himba individuals were included in this sleep analysis, with older age predicting longer sleep duration and higher sleep efficiency. This finding supports the idea that short sleep in this population and gender differences in duration and efficiency may be the result of high levels of evening socialization, especially in men and younger individuals [44]. Overall, it appears that socio-ecological contexts may evoke gendered effects in sleep-wake regulation.

Bolivian Tsimane forager-horticulturalists exhibit highly variable sleep onset timing that is associated with social and work activities which differ by occupation and between men and women, such as hunting and fishing; these activities were found to contribute to a decrease in total sleep time of 1.3 and 2.32 hours, respectively [45]. Tsimane women’s sleep duration is on average longer than men’s, a pattern which is attributed in part to differential labor demands [45]. Similarly, it appears that a combination of labor and social demands is contributing to men’s decreased sleep duration in the Maya community as well, where men also appear to be involved in more nighttime social activity than women.

The interaction between gender and age suggests that age predicts longer sleep duration in women, but shorter sleep duration in men (Fig 2 and S3 Table). It is surprising that age was negatively associated with sleep duration in men, as we expected younger men to spend more time participating in late-night social activities that contribute to shorter sleep duration and more variation in bedtime and wake time. It is possible that the younger men in our sample, some of whom are in their late teens and early 20s, are more constrained by household rules and therefore limited in how late they are allowed to stay out, while older men many be more likely to have the freedom to stay out later at night. Furthermore, our results add to the consistent pattern of men showing a more evening-preferring chronotype than women, perhaps due to a possible benefit to social networking [26,28], as well as cultural norms and prescriptive social rules that limit women’s nighttime socializing. Our findings help to clarify gender differences in sleep patterns and speak to the importance of considering cultural factors, such as gender expectations, labor roles, and social activities.

For men in particular, supplemental agriculture work on weekends may in part explain Maya sleep durations that were shorter than those in industrial populations, where longer sleep on weekends and free days could contribute to higher population averages. Other weekend morning activities that are common in this Maya community, such as visiting the market or attending church, could influence sleep duration as well. Specific activity patterns should be studied in future work for a more complete understanding of how Maya economic and social activities may be contributing to the relatively shorter sleep durations observed in our analysis.

### Limitations

There are limitations to our study which warrant attention. First, we lack quantitative data on the number of households with access to electricity, home electric devices, and information on the frequency of power outages. While access to electricity and ownership of home electric devices such as televisions and refrigerators has increased over the past two decades, there is still the possibility that individual and household variation in the use of electricity could play a role in sleep and rest-activity patterns. Future research should employ further quantitative measurement of these factors.

A second limitation involves the comparability of our comparative samples. The data for our comparisons between Maya, Malagasy, and MIDUS samples were generated using CamNtech MotionWatch 8 actigraphs, collected in 60-second intervals (Maya and Malagasy), and Mini Mitter Actiwatch-64 activity monitors, collected in 30-second intervals (MIDUS). Sleep data scoring protocols corresponded to their respective accelerometers, and data cleaning and analysis was done by different individuals, which introduces possible rater inconsistencies. However, we addressed these limitations to the best of our ability by using comparative sleep variables that were generated with high consistency in scoring protocols. Differences in 60-second intervals vs 30-second intervals have been previously found to be insignificant in the generated actigraphy results [51]. A number of other socio-ecological factors could contribute to differences in sleep and rest-activity patterns that are difficult to account for in our comparative analyses between these samples as well. For example, differences in season and latitude in which data were collected, which can affect sleep patterns through temperature and sunlight exposure, could be partly responsible for some of the differences between populations that we report here.

Finally, a potential limitation concerns the chronotype results. Sleep mid-point on free days, when bedtime and waketimes are not constrained by work responsibilities, would more accurately serve as a proxy for individual’s sleep patterns and chronotype. However, we do not have data available on participants’ work schedules, and are therefore limited in our conclusions regarding the relative contribution of physiology and cultural factors in influencing chronotype patterns reported here.

### Conclusions

In sum, sleep data from this Maya community that is currently undergoing an economic transition which affects zeitgeber exposure helps in furthering our understanding of the environmental, economic, and cultural drivers of human sleep. Our results are consistent with those of other recent studies suggesting that, despite the sleep disruptive effects of electricity on sleep, industrialization does not inherently drive significant reductions in sleep duration and efficiency compared to nonindustrial, or “natural” human sleep (e.g., [8,16,52]). In the cultural West, there is evidence suggesting harmful effects of artificial lighting on sleep patterns. Yet, it appears that sleep in the Mayan sample—which was longer and more efficient than sleep in many small-scale populations with less industrialization—benefits from the safety and comfort of sleeping sites that are relatively buffered from the environment. Thus, these findings suggest that despite arguments implicating urbanization in giving rise to a sleep loss epidemic [14,15], the climate control and safety of urban settings may contribute to longer, better quality sleep while paradoxically also be leading to circadian rhythm weakening and desynchronization through decreased exposure to these zeitgebers [16]. For individuals living in industrialized environments, our results suggest that intentional exposure to natural sunlight, minimizing use of electrical devices around bedtime, and maintaining consistent bedtime and wake schedules can improve sleep health and general wellbeing.

## Supporting information

S1 Table Results from linear mixed effects models predicting sleep duration, efficiency, and WASO between Maya and MIDUS cohorts. Significance codes: * p < 0.05; ** p < 0.01; *** p < 0.001.
(PDF)

S2 Table Results from linear regression models predicting interdaily stability and intradaily variability between Maya and MIDUS cohorts. Significance codes: * p < 0.05; ** p < 0.01; *** p < 0.001.
(CSV)

S3 Table Results from linear mixed effects model predicting sleep duration. Male is the reference gender. Significance codes: * p < 0.05; ** p < 0.01; *** p < 0.001.
(CSV)

S4 Table Results from linear mixed effects model predicting sleep efficiency. Male is the reference gender. Significance codes: * p < 0.05; ** p < 0.01; *** p < 0.001.
(CSV)

S5 Table Results from linear mixed effects model predicting central phase. Male is the reference gender. Significance codes: * p < 0.05; ** p < 0.01; *** p < 0.001.
(CSV)
