# Supplementary material for: Technological infrastructure, sleep, and rest-activity patterns in a Kaqchikel Maya community
Source: PLoS One. 2022 Nov 16;17(11):e0277416. doi: 10.1371/journal.pone.0277416 (PMC9668134; doi:10.1371/journal.pone.0277416)
Supplement: S1 Table — Significance codes: * p < 0.05; ** p < 0.01; *** p < 0.001. (PDF) [file pone.0277416.s001.pdf]

S1 Table. Results from linear mixed effects models predicting sleep duration, efficiency, and WASO between Maya and MIDUS cohorts.

	**Time in bed**	**Sleep latency**	**Sleep duration**	**Sleep efficiency**	**WASO**
	Estimate (SE)	*Estimate (SE)*	*Estimate (SE)*	*Estimate (SE)*	*Estimate (SE)*
Cohort (ref=Maya)	-0.26 (0.04)***	0.12 (0.04)**	0.001 (0.05)	0.19 (0.05)***	-0.51 (0.04)***
Gender (ref=female)	-0.14 (0.03)***	0.10 (0.03)**	-0.19 (0.04)***	-0.19 (0.04)***	0.07 (0.03)*
Age	-0.09 (0.03)**	0.02 (0.03)	-0.08 (0.04)*	-0.02 (0.04)	-0.05 (0.03)

Significance codes: * $p < 0.05$; ** $p < 0.01$; *** $p < 0.001$.

S2 Table. Results from linear regression models predicting interdaily stability and intradaily variability between Maya and MIDUS cohorts.

	**Relative Amplitude**	**Interdaily Stability**	**Interdaily Variability**
	Estimate (SE)	*Estimate (SE)*	*Estimate (SE)*
Cohort (ref=Maya)	-0.004 (0.01)	-0.15 (0.02)***	0.07 (0.03)*
Gender (ref=female)	-0.02 (0.01)*	-0.03 (0.01)*	-0.003 (0.02)
Age	-0.001 (0.0003)	0.001 (0.001)	0.001 (0.001)

Significance codes: * $p < 0.05$; ** $p < 0.01$; *** $p < 0.001$.

S3 Table. Results from linear mixed effects model predicting sleep duration.

**Predictor**	**Estimate (standard error)**	**Confidence interval**	**Importance**	**P-value**
Age	-0.50 (0.19)	-0.87 - -0.13	2nd = 0.92	0.008 **
Gender	0.27 (0.12)	0.03 - 0.51	1st = 0.93	0.027 *
Rainfall	-0.09 (0.04)	-0.17 - -0.002	4th = 0.73	0.046 *
Humidity	0.07 (0.05)	-0.03 - 0.16	5th = 0.49	0.159
Temperature	0.06 (0.04)	-0.03 - 0.15	6th = 0.47	0.183
Age*Gender	0.56 (0.16)	0.24 - 0.88	3rd = 0.89	<0.001 ***

Male is the reference gender. Significance codes: * $p < 0.05$; ** $p < 0.01$; *** $p < 0.001$.

S4 Table. Results from linear mixed effects model predicting sleep efficiency.

Predictor	Estimate (standard error)	Confidence interval	Importance	P-value
Age	0.16 (0.17)	-0.17 - 0.48	1st = 0.89	0.347
Gender	-0.003 (0.13)	-0.25 - 0.25	3rd = 0.51	0.978
Rainfall	-0.06 (0.04)	-0.14 - -0.02	2nd = 0.52	0.136
Humidity	0.02 (0.04)	-0.07 - 0.11	6th = 0.29	0.646
Temperature	0.06 (0.04)	-0.02 - 0.14	4th = 0.49	0.163
Age*Gender	0.30 (0.17)	-0.03 - 0.64	5th = 0.29	0.077

Male is the reference gender. Significance codes: * $p < 0.05$; ** $p < 0.01$; *** $p < 0.001$.

S5 Table. Results from linear mixed effects model predicting central phase.

**Predictor**	**Estimate (standard error)**	**Confidence interval**	**Importance**	**P-value**
Age	-0.23 (0.21)	-0.65 - 0.19	3rd = 0.52	0.279
Gender	-0.32 (0.14)	-0.59 - -0.06	1st = 0.95	0.018 *
Rainfall	-0.03 (0.03)	-0.09 - 0.04	5th = 0.32	0.453
Humidity	0.06 (0.04)	-0.01 - 0.13	2nd = 0.55	0.120
Temperature	0.03 (0.03)	-0.03 - 0.10	4th =0.37	0.332
Age*Gender	0.33 (0.20)	-0.06 - 0.73	6th = 0.27	0.099

Male is the reference gender. Significance codes: * $p < 0.05$; ** $p < 0.01$; *** $p < 0.001$.
